# Gene expression analysis to detect disseminated tumor cells in the bone marrow of triple-negative breast cancer patients predicts metastatic relapse

**DOI:** 10.1007/s10549-019-05405-7

**Published:** 2019-08-20

**Authors:** Chidananda M. Siddappa, Sreeraj G. Pillai, Jackie Snider, Patsy Alldredge, Kathyrn Trinkaus, Mark A. Watson, Rebecca Aft

**Affiliations:** 1 Department of Surgery, Washington University School of Medicine, Box 8109, 660 South Euclid Avenue, St. Louis, MO 63110 USA; 2 Department of Pathology and Immunology, Washington University School of Medicine, St. Louis, MO USA; 3 Division of Public Health Science, Department of Surgery, Washington University School of Medicine, St. Louis, MO USA; 4 Siteman Cancer Center, Washington University School of Medicine, St. Louis, MO USA; 5 John Cochran Veterans Administration Hospital, St. Louis, MO USA

**Keywords:** Triple-negative breast cancer, Disseminated tumor cells, Metastasis, Gene expression, Targeted therapy

## Abstract

**Purpose:**

Disseminated tumor cells (DTCs) in the BM of breast cancer patients predict early disease relapse, but the molecular heterogeneity of these cells is less well characterized. Expression of a 46-gene panel was used to detect DTCs and classify patient BM samples to determine whether a composite set of biomarkers could better predict metastatic relapse.

**Methods:**

Using a high-throughput qRT-PCR assay platform, BM specimens collected from 70 breast cancer patients prior to neoadjuvant therapy were analyzed for the expression of 46 gene transcripts. Gene expression was scored positive (detectable) relative to a reference pool of 16 healthy female control BM specimens. To validate findings from a subset of 28 triple-negative breast cancer (TNBC) patients in the initial 70 patient cohort, an independent set of pre-therapeutic BM specimens from 16 TNBC patients was analyzed.

**Results:**

Expression of each of the 46 gene transcripts was highly variable between patients. Individual gene expression was detected in 0–84% of BM specimens analyzed and all but two patient BM specimens expressed at least one transcript. Among a subset of 28 patients with TNBC, positivity of one or more of eight transcripts correlated with time to distant relapse (*p* = 0.03). In an independent set of 16 triple-negative patient BM samples, detection of five of these same eight gene transcripts also correlated with time to distant relapse (*p* = 0.03) with a positive predictive value of 89%.

**Conclusions:**

We identified a set of gene transcripts whose detection in the BM of TNBC patients, prior to any treatment intervention, predicts time to first distant relapse, thus identifying a TNBC patient population which requires additional treatment intervention. Because these genes are presumably expressed in populations of DTCs and many encode proteins that are known therapeutic targets (e.g., ERBB2), these results also suggest a potential approach for targeted DTC therapy to mitigate distant metastases in TNBC.

**Electronic supplementary material:**

The online version of this article (10.1007/s10549-019-05405-7) contains supplementary material, which is available to authorized users.

## Introduction

Metastasis is the most significant contributor to mortality in breast cancer patients. Many preclinical studies suggest that only a small, unique subset of cells within a primary tumor possesses metastatic potential and that the molecular phenotype of these cells may evolve as they transition outside of the primary tumor and progress to metastatic foci [1–3]. To develop new therapeutic interventions that will effectively monitor and prevent overt distant disease development, it is essential to identify and target these intermediary cells in the metastatic process since these cells likely have biological behavior and therapeutic vulnerabilities which differ from the corresponding primary tumor.

Disseminated tumor cells (DTCs) can be detected in the bone marrow (BM) of 30–40% of early-stage breast cancer patients by immunocytochemical (ICC) staining of cytokeratins. Several large multi-institutional clinical studies have documented that BM DTCs are an independent prognostic factor for patients with stage I–III disease [4–6]. While the detection of BM DTCs may simply be a surrogate marker of body-wide tumor cell dissemination, an attractive alternative hypothesis is that BM serves as a specific reservoir that allows DTCs to adapt and disseminate to other organs after some period of latency [7].

DTCs are molecularly heterogeneous, genetically distinct from their originating primary tumor, and likely have varying metastatic potential [8–10]. They can persist for years and remain a predictor of disease recurrence [11]. Patients with detectable DTCs in their BM after chemotherapy have a particularly poor prognosis, suggesting that conventional chemotherapy does not eliminate all DTCs and that those subpopulations which survive cytotoxic chemotherapy have a high metastatic potential and may exhibit a stem cell-like chemotherapy-resistant phenotype [12].

Although DTCs may be tenfold more abundant than circulating tumor cells (CTCs) found in the peripheral blood, they are still exceedingly rare (1–10 DTCs per million mononuclear immunocytes) which makes their individual molecular characterization difficult [13]. Current methods to detect BM DTCs by ICC or single-gene expression biomarkers are technically demanding and lack sufficient sensitivity, specificity, and clinical utility for use in routine clinical practice, particularly where serial monitoring may be required [14, 15]. Therefore, the development of new analytical techniques and biomarkers for the detection, classification, and, in particular, therapeutic targeting of BM DTCs could identify patients at high risk for the development of distant metastatic disease and mitigate its progression.

We previously developed a strategy for identifying genes whose expression is associated with BM DTCs in breast cancer patients [16, 17] and now describe a sensitive, multiplex gene expression assay platform (Fluidigm Biomark HD) for their detection. The current study demonstrates the potential clinical utility of a 46-gene expression panel that detects and classifies BM DTCs in breast cancer patients. More specifically, in ‘triple-negative’ breast cancer (lacking expression of estrogen receptor and Her2, TNBC) patients, expression of an eight-gene subset of this panel correlates with time to distant metastatic relapse in two independent cohorts of patients. Since many of these genes and their corresponding proteins are actionable therapeutic targets, in addition to risk-stratification, this also suggests a new paradigm for the detection and targeted elimination of BM DTCs based upon specific and unique molecular markers, to prevent metastatic progression.

## Materials and methods

### Patients

Under an IRB-approved protocol (# 201309087) with informed consent, anonymized control BM specimens were collected from female volunteers with no current or past history of cancer, who were undergoing hip replacement surgery. BM specimens for the test clinical cohort were collected from an ongoing IRB-approved BM and tissue banking protocol from clinical stage II/III breast cancer patients at our institution which began in 2011 (IRB # 201101961). BM specimens for the validation cohort of clinical stage II/III TNBC patients were obtained from a retrospective collection banked in the context of a previous clinical trial evaluating the use of zoledronic acid to prevent growth and survival of DTCs (NCT00242203) [18]. Only patients in the control arm, not receiving zoledronic acid, were evaluated in this study. Patients received standard chemotherapy with an anthracycline, cyclophosphamide and a taxane. Patients with Her2-positive tumors received Trastuzumab and estrogen receptor (ER)-positive patients received adjuvant hormonal therapy. All patient BM aspirates used for the current study were collected prior to the initiation of any therapeutic interventions.

### BM aspiration, processing, and RNA isolation

For all breast cancer cases, 20 ml of BM was collected from both the right and left anterior iliac crests, subjected to hypotonic RBC lysis, washing, and nucleated cell counting within 2 h from the time of collection. For controls, BM was harvested unilaterally. For whole BM analysis, 5 × 10^6^ nucleated cells were pelleted and immediately snap frozen for subsequent RNA isolation. All RNA isolations were performed from snap-frozen cell pellets using Trizol reagent (Invitrogen). RNA was quantified and qualitatively assessed by A260, A280, and A230 readings using a Nanodrop spectrophotometer, and further qualified by RIN score using an Agilent bioanalyzer. For breast cancer samples, 500 ng from each left and right BM sample was pooled for a single sample analysis from each patient in the test cohort. In the validation cohort, left and right BM samples were analyzed independently and gene expression positivity in either one of the sample pairs was considered positive for the patient.

### Multiplexed qRT-PCR

Quantitative PCR was performed using a microfluidic-based PCR system with 96.96 Dynamic Arrays (Fluidigm Corp. San Francisco, CA). For each BM sample, cDNA was synthesized from 50 ng of total RNA using Life Technologies High-Capacity cDNA Reverse Transcription Kit (Applied Biosystems, Foster City, CA). Each cDNA was then subjected to specific target amplification (STA) using TaqMan PreAmp Mastermix (Applied Biosystems, Foster City, CA). The samples underwent 14 rounds of amplification in the STA process. The cycling program consisted of 10 min at 95 °C followed by 14 cycles of 95 °C for 15 s and then 60 °C for 1 min. Each completed STA reaction was diluted 1:4 in low-EDTA DNA suspension buffer for qPCR. Reactions were prepared by combining samples with TaqMan Universal PCR Master Mix (Applied Biosystems) and 20× Gene Expression Sample Loading Reagent (Fluidigm Corp.). The assay mixtures contained 9 µM of each primer and 2 µM of the probe in Dynamic Array Assay Loading Reagent (Fluidigm Corp). Primer and probe sequences for each transcript used for the STA and PCRs are listed in Supplemental Table 1. Sample and assay mixtures were loaded into the primed 96.96 Dynamic Array chip and the NanoFlex-4 Integrated Fluidic Circuit Controller was used for the distribution of the sample and assay mixture. The loaded Dynamic Array was run on a BioMark™ Reverse-Transcription-PCR System. The qPCR program was as follows: 50 °C for 2 min, 95 °C for 2 min, 40 cycles of 95 °C for 15 s and 60 °C for 1 min. All PCRs were run in duplicate.

### Data and statistical analysis

Each of the 9216 PCRs in the array produced a raw PCR cycle threshold (CT) value which was used for subsequent calculation. Duplicate PCRs which deviated by more than 1 CT were discarded and marked as ‘failed’. Expression of each of the 46 target genes in each control sample was calculated using the ddCT method, with expression of *GUSB* used as the normalizer for each sample. Since the expression of most target genes (by definition) was not detected in control BM samples, a baseline of 27 cycles was defined as ‘not detected’. The average and standard deviation (SD) of expression of each gene across all control samples were calculated. This process was repeated for each of the patient samples. When the relative expression level of a gene in a patient sample exceeded 3 SD above the mean of the control samples, expression of that gene was scored as positive. Hierarchical clustering and principal component visualizations were performed using binary data (‘positive’ vs. ‘negative’) and Partek Genomics Suite. Time to distant relapse was defined as the time from diagnosis to the time of first-detected distant recurrence. Kaplan–Meier plots were generated with Graph Pad Prism.

## Results

### Molecular profiling of BM samples

To better understand the clinical relevance of a previously identified DTC-associated gene expression panel, which comprised a set of 46 genes frequently expressed by breast cancer cells but with very limited or absent expression in healthy BM samples [17], we evaluated expression in the pre-therapeutic BM of 70 clinical stage II/III breast cancer patients using high-density array-based qRT-PCR. Twenty of the 70 patients in this cohort (29%) developed distant metastatic relapse (Table 1). The largest percentage of recurrences was observed in the TNBC patients (42%) and the ER+/Her2+ (45%). Among the 70 patients, 69 had detectable expression of one or more of the DTC panel genes. The patient specimen lacking in expression of any of the genes was Her2 positive and did not develop metastases. The total number of genes expressed ranged from 1 to 23 in each patient’s BM but was unrelated to risk or time to distant disease relapse (data not shown).

**Table 1:** Clinical characteristics 70 patients evaluated for the expression of 46 genes

	Recurrent (*n* = 20)	Non-recurrent (*n* = 50)
Mean age (years)	49	50
Race		
Caucasian	13	32
African American	7	18
Clinical stage		
I	0	1
II	7	26
III	7	7
Average tumor size (cm)		
Pretreatment	5.9	3.7
Post-treatment	4.7	1.6
pCR	1	15
Histology		
IDC	17	46
IMC	2	3
Grade		
I	0	3
II	4	15
III	16	32
Biomarkers		
ER+/HER2−	3	20
ER+/HER2+	5	6
ER−/HER2+	0	8
TN	12	16

**Fig. 1:**
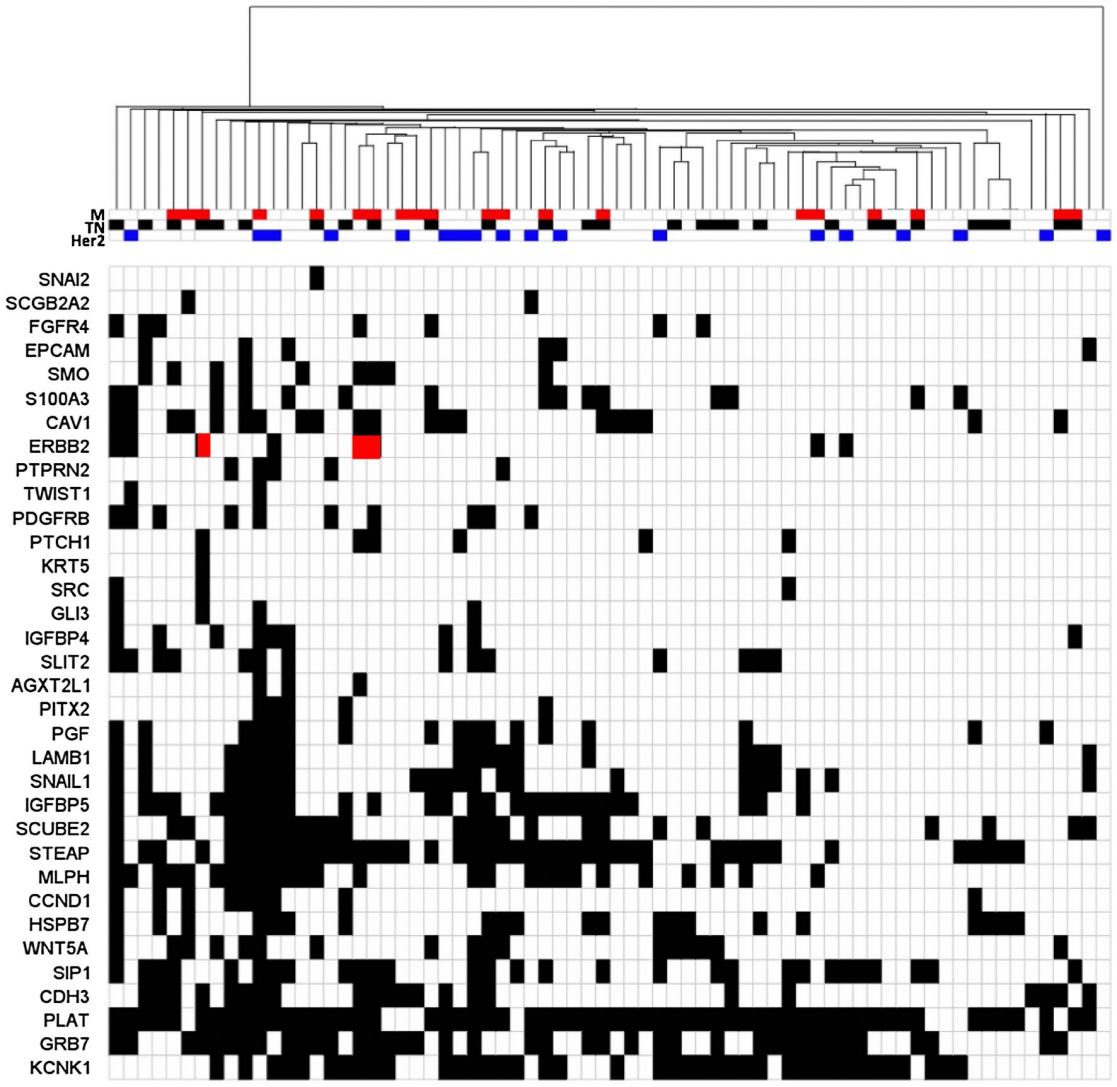
Hierarchical cluster visualization of 34 expressed gene transcripts, across 70 BM samples from breast cancer patients. Patients with metastatic relapse (M), patients with triple-negative disease (TN), and patients with HER2-positive primary tumors are shown in the top annotation tracks. Patients with *ERBB2*-positive BM samples and HER2-negative primary tumors, who experienced metastatic relapse are highlighted in red

Among individual genes, 12 of the 46 genes (*CLDN4, EGFR, ESR1, FOXA1, HES1, KRT17, KRT19, LOXL2, MAGEA3, MAPT, NPY1R* and *PTEN*) in the panel were not detected in any of the patient BM samples. In some cases, such as KRT19, gene expression was detectable but levels were not above those detected in the BM of the reference female heathy controls, supporting previous studies that show this marker lacks specificity [14]. The remaining genes were expressed in 1–23 patient BM samples. Individually, the detection of several genes was associated primary tumor ER status (*GRB7, SCUBE2, LAMB1, WNT5A, EPCAM*), lymph node status at presentation (*STEAP 1*), and, in particular, distant metastatic relapse (*MLPH, AGXT2L1*), although none of these associations was significant after correction for multiple comparisons (Supplemental Table 2). Of note, EpCAM, which is frequently used as a target for enrichment for DTCs was expressed in 8% of the samples. As shown in Fig. 1, the overall expression pattern of the 46 genes was extremely heterogeneous and no subsets of genes demonstrated coordinated expression between patient BM specimens.

### BM gene expression of actionable biomarkers

**Fig. 2:**
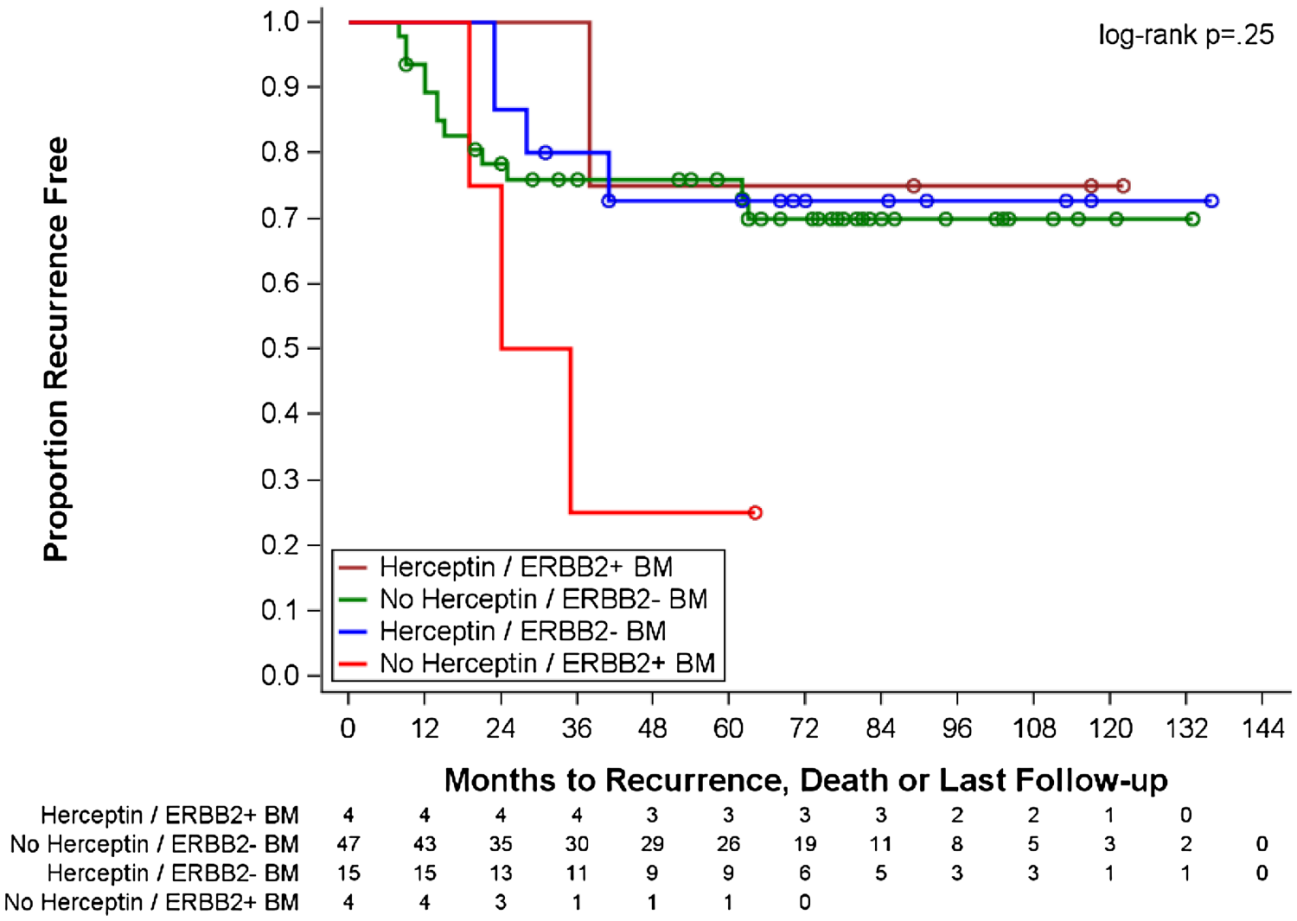
Kaplan–Meier analysis of time to metastatic relapse for 70-patient cohort, stratified by *ERBB2* detection in BM and whether the patient received Trastuzumab therapy for a corresponding ERBB2-positive primary tumor

Because several of the genes in the panel were purposely chosen for their targetable therapeutic relevance (e.g., ERBB2, PDGFRB, STEAP1, WNT5A), it was particularly interesting to note recurrent, detectable expression of these genes in many patient BM samples. For example, as previously noted by others [19, 20], we detected *ERBB2* expression in four patient BM samples, although the corresponding primary tumor was *ERBB2* negative (Fig. 1). Although the presence of *ErbB2* expression in the BM of *ErbB2* (Her2)-positive patients had no impact on progression to distant metastases, as shown in Fig. 2, patients with *ErbB2*-negative tumors who were not treated with Trastuzumab had a much shorter time to metastatic recurrence when there was detectable erbB2 expression in BM (HR 3.0, 95% CI 0.85–10.62). Although the number of such patients in this analysis was small, it suggests the utility of biomarker-directed targeted therapy based on BM DTC molecular profiling in pre-metastatic patients, independent of primary tumor status. In fact, this is a paradigm that has been now tested for BM *erbB2* expression as a predictive marker in a clinical trial setting (NCT01779050), currently in follow-up.

### Prognostic utility of BM gene expression

**Fig. 3:**
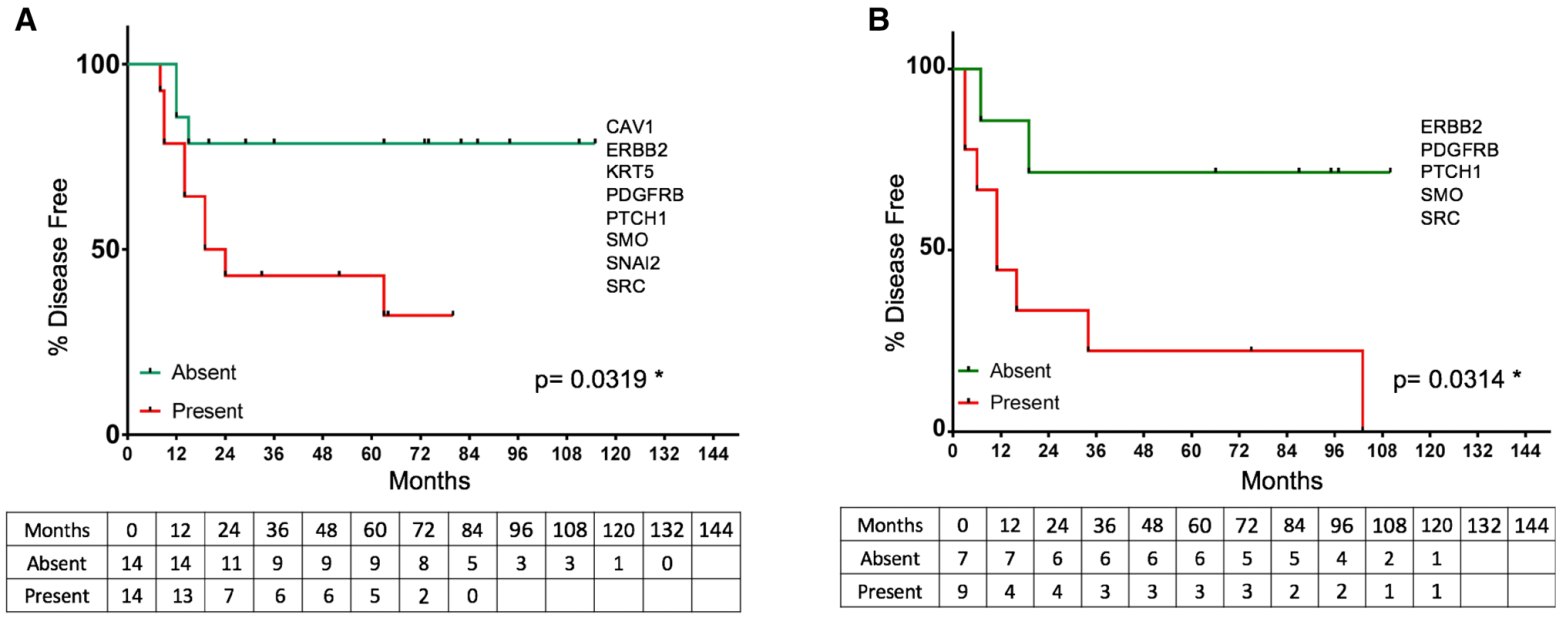
Kaplan–Meier analysis of time to distant metastatic relapse for TNBC patients based upon gene expression. **a** Initial 28 patients using the 8-gene expression panel; **b** independent, validation cohort of 16 patients using the 5-gene expression subpanel

**Fig. 4:**
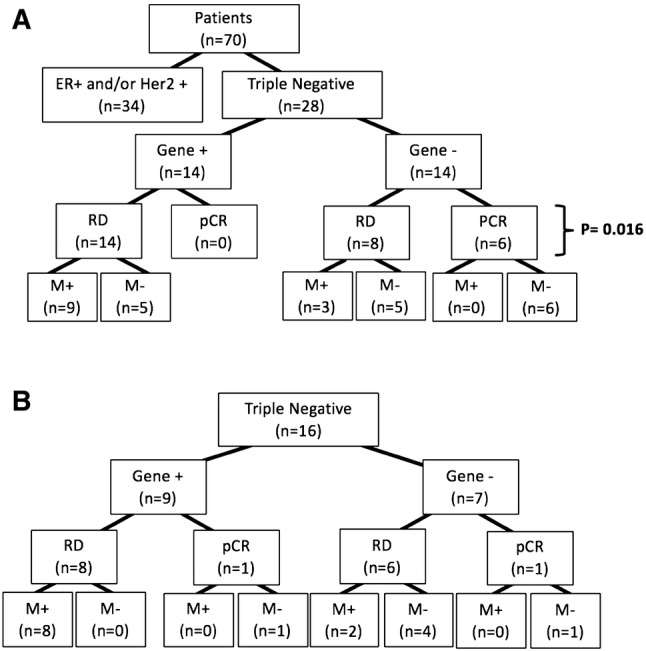
Distribution of initial 70-patient test cohort, based on primary tumor molecular phenotype, 8-gene expression panel result at initial diagnosis, subsequent pathological response to neoadjuvant therapy, and eventual progression to distant metastasis. Correlation between gene expression panel result and residual disease is shown (Fisher’s exact test). *RD* residual disease, *pCR* pathological complete response, *M* distant metastatic disease development

As expected, detectable BM gene expression was not strongly prognostic based upon single-gene biomarkers (Supplemental Table 2). We, therefore, attempted to define subsets of genes whose combined expression in pre-therapeutic BM samples could collectively identify those patients at highest risk for distant metastatic relapse. No combination of gene expression was able to distinguish outcomes among all 70 patients. Therefore, to address a more clinically homogenous cohort with frequently aggressive disease, we focused specifically on a subset of 28 patients with ‘triple-negative’ (ER negative, progesterone receptor negative, Her2 negative, TNBC) primary tumors. After optimizing a minimal combination of genes whose expression produced the greatest sensitivity and specificity for distant disease recurrence, we identified a panel of eight genes—*KRT5, SNAI2, PTCH1, CAV1, SMO, ERBB2, PDGFRB*, and *SRC*. TNBC patients whose BM had detectable levels of at least one of these eight genes had significantly shorter time to distant metastatic relapse than patients whose BM had no detectable expression of any of these genes (*p* = .0319) (Fig. 3a). Additionally, those patients whose pre-therapeutic BM panel was positive for at least one gene never experienced a complete pathological response to neoadjuvant therapy, as compared to those patients who had no detectable BM gene panel expression prior to treatment (Fig. 4a). Among these eight genes, the specific combination and number of detectable transcripts itself were unrelated to outcome. This same eight-gene expression panel was also not prognostic for time to progression in patients with ER-positive or Her2-positive tumors. Although the total number of patients and percent recurrence was limiting in this subset analysis, this result suggests that the biomarker panel may be specific for predicting progressive disease only in triple-negative patients.

Because of the obvious ‘over-fitting’ of the gene expression panel in the first sample set, we next evaluated the prognostic significance of the same 8-gene BM expression panel in a second, independent cohort of 16 patients with TNBC (Table 2). Pre-therapeutic BM samples from these patients were obtained in the context of a therapeutic trial of zoledronic acid administration [18]. Although the eight-gene expression panel lacked sufficient specificity to fully predict distant metastatic relapse in this cohort using the same criteria, a set of five of the same eight genes (*ERBB2, PDGFRB, PTCH1, SMO,* and *SRC*) analyzed in this cohort also significantly stratified those patients into low and high risks for distant metastatic relapse (*p* = .0314, Fig. 3b).

**Table 2:** Clinical characteristics 16 TNBC patients (validation cohort) evaluated for 8-gene BM DTC signature

	Recurrent (*n* = 10)	Non-recurrent (*n* = 6)
Mean age (years)	44	53
Race		
Caucasian	4	4
African American	5	2
Clinical stage		
I		
II		
III		
Average tumor size (cm)		
Pretreatment	3.8	2.7
Post-treatment	3.8	1.6
pCR	0	2
Histology		
IDC	9	6
IMC	1	0
Grade		
I	0	0
II	1	0
III	9	6

## Discussion

The presence of DTCs in the BM of early-stage breast cancer patients has demonstrated prognostic significance [4–6]. Most assays employ ICC detection of epithelial antigens [14] or PCR-based gene expression [15] but have not been adopted as standard-of-care diagnostics because BM aspirations are not routinely performed on breast cancer patients, current detection assays are technically complex or expensive, and results are not immediately actionable in most breast cancer patients. The present study was designed to address whether multi-marker detection of DTCs and molecular classification of BM could result in a more specific, sensitive, robust, and actionable assay.

The 46-gene expression panel described in this report was originally derived from a series of microarray-based gene expression experiments to define ‘DTC-specific’ gene expression [16]. This gene list was refined based upon absent expression in panels of age-matched BM samples from healthy female volunteers and detectable expression in a number of breast cancer cell lines and primary tumors [17]. The gene list was also purposefully biased toward selection of genes which could serve as potential therapeutic targets. In the absence of single-cell expression analyses, it is not definitively known whether the expression of each gene originates from tumor cells themselves or reactive, rare BM cell populations, specifically in patients with micrometastatic disease. The precise cellular origin of gene expression is an interesting biological question that is the topic of ongoing studies using single-cell expression profiling and in situ gene expression analyses. However, preliminary data from our laboratory, using RNA in situ hybridization, confirm the expression of *ERBB2* and *STEAP1* by DTCs. Several other previously defined DTC gene expression biomarkers [21], particularly related to ‘stem-cellness’, were not included in this 46-gene panel, primarily because they are frequently detected in the BM of non-breast cancer patients and lacked the stringent specificity required for the assay.

It was surprising to find both the high frequency and great heterogeneity of gene expression in the BM samples from this patient cohort. We found the expression of at least one of the 46 genes was detected in all but one Her2-positive patient, whereas the traditional detection of DTCs by ICC methods averages 20–30% in similarly staged breast cancer patients [5, 6]. One explanation for this discrepancy may be that traditional immunocytochemistry methods only measure epithelial antigens such as cytokeratin and EpCAM, which can be frequently downregulated in metastatic tumor cells or cells that revert to a ‘stem cell-like’ phenotype [14, 15]. For example, in our BM cohort, *EpCAM* expression was detected in only 8% of patient BM and *Keratin19* was not significantly expressed in any of the specimens. In fact, the prevalence of BM DTCs in stage II/III breast cancer may be much higher when a broader gene expression-based biomarker assessment is considered, even if DTCs detected by this approach do not all have uniform metastatic potential. This would be consistent with the continued release of thousands of cancer cells from the primary tumor.

Our initial hypothesis was that a set of identifiable genes would be detectable in all DTCs regardless of tumor subtype which allows them to migrate and survive in the BM. However, the overall heterogeneity of gene expression observed was not associated with sample clustering based upon patterns of gene expression and there was no significant association with ER status or tumor stage. This could in part be due to technical noise since the qRT-PCR assay itself is purposed to detect gene expression in very rare populations of cells, at a very low and nearly indistinguishable level above background. Adaptation of new technologies using sample pre-amplification and droplet digital PCR could address this current limitation. Alternatively, the heterogeneity observed could be biologically meaningful and simply reflect the heterogeneity of patients and primary tumors themselves. This is further suggested by the fact that when one specific patient subpopulation (triple-negative disease) was examined, a much more uniform and prognostic pattern of gene expression was observed. The fact that the eight-gene signature identified in the first cohort could be validated, at least in part, in a second independent cohort suggests that this pattern of gene expression is more uniform and generalizable among triple-negative patients with BM DTCs. However, the fact that only five of the originally eight genes chosen could provide prognostic accuracy also suggests that further studies with additional patients and more sensitive detection techniques will be necessary.

In TNBC, pathologic complete response (pCR) after neoadjuvant therapy is a significant predictor of survival and distant relapse [22, 23]. In this study, gene expression-based DTC assessment was performed in BM samples obtained prior to any neoadjuvant therapy, purposely to avoid any confounding changes in gene expression in the BM due to chemotherapeutic effect. Detection and classification of DTCs in treatment-naïve patients was comparable to (in the first cohort) and superior to (in the second cohort) assessment of primary tumor pCR after neoadjuvant therapy for predicting time to distant metastases (*p* = 0.05 vs. *p* = 0.03 in cohort 1; *p* = 0.02 vs. *p* = 0.12 in cohort 2, Fisher’s exact test). This might be unexpected as therapeutic course could greatly influence the residual DTC population and affect overall and recurrence-free outcomes, although the TNBC patients in this study received fairly uniform neoadjuvant and adjuvant therapeutic regimens. Importantly, it is also interesting to note that a positive eight-gene DTC panel result, assayed prior to the initiation of any therapy, also correlated with primary tumor response to neoadjuvant therapy (Fig. 4). In this regard, with respect to TNBC patients, it is possible that those same patients with early tumor cell dissemination to the BM or very heterogeneous tumors may also be those whose primary tumors and DTCs are more refractory to conventional chemotherapy. It will be of future interest to compare patterns of BM DTC gene expression pre- and post-therapy to determine whether changes in gene expression are more clinically significant for predicting future metastatic spread.

Finally, beyond the potential prognostic utility of this gene expression panel, this study demonstrates a possible approach to BM-based targeted therapeutics. As previously reported [20], patients may frequently present with ERBB2-negative tumors (who were not treated with Trastuzumab) but with detectable *ERBB2*-positive gene expression in their BM, presumably emanating from DTCs. In this study, four such patients had a much shorter time to distant metastatic relapse as compared to both patients with no detectable DTCs and importantly, patients with *ERBB2*-positive DTCs who were treated with Trastuzumab because their primary tumor was also ERBB2 positive. Several other ‘DTC-specific’ genes in this panel (PDGFRB, STEAP1, SRC, WNT5A, PTCH1), although not necessarily prognostic in this study, could also serve as potential therapeutic targets, even if they have proven less effective targets for the treatment of primary breast tumors in otherwise unselected patient population [24, 25]. Given that the expression of these genes is sufficiently specific, we foresee a potential to use this panel for the identification of patients who are at high risk of distant recurrent disease development despite standard-of-care chemotherapy and who may benefit from biomarker-directed therapy based on DTC (rather than primary tumor) gene expression to eradicate BM DTCs and mitigate future metastatic relapse in breast cancer patients.

## Electronic supplementary material

Below is the link to the electronic supplementary material.

Supplementary material 1 (DOCX 26 kb)
