# Supplementary material for: Gene expression analysis to detect disseminated tumor cells in the bone marrow of triple-negative breast cancer patients predicts metastatic relapse
Source: Breast Cancer Res Treat. 2019 Aug 20;178(2):317–25. doi: 10.1007/s10549-019-05405-7 (PMC6797655; doi:10.1007/s10549-019-05405-7)
Supplement: Supplementary file 1 — Supplementary material 1 (DOCX 26 kb) [file 10549_2019_5405_MOESM1_ESM.docx]

| **Gene symbol** | **Taqman Primer** | **Gene symbol** | **Taqman Primer** |
| --- | --- | --- | --- |
| CAV1 | Hs00971716_m1 | PLAT | Hs00263492_m1 |
| CCND1 | Hs00765553_m1 | PTCH1 | Hs00181117_m1 |
| CDH3 | Hs00999925_m1 | PTPRN2 | Hs01109231_m1 |
| CLDN4 | Hs00976831_s1 | S100A3 | Hs01112771_g1 |
| EGFR | Hs01076078_m1 | SCGB2A2 | Hs00935948_m1 |
| EPCAM | Hs00901885_m1 | SCUBE2 | Hs01012962_m1 |
| ERBB2 | Hs01001580_m1 | SIP1 | Hs01026708_g1 |
| ESR1 | Hs00174860_m1 | SLIT2 | Hs01061399_m1 |
| FGFR4 | Hs00608743_g1 | SMO | Hs01090242_m1 |
| FOXA1 | Hs04187555_m1 | SNAI2 | Hs00950344_m1 |
| GLI3 | Hs00609233_m1 | SNAIL1 | Hs00195591_m1 |
| GRB7 | Hs00917999_g1 | SRC | Hs01082246_m1 |
| GUSB_REF | Hs00939627_m1 | STEAP | Hs00185180_m1 |
| HSpb7 | Hs00205296_m1 | TBP_REF | Hs00427620_m1 |
| IGFBP4 | Hs01057900_m1 | TWIST1 | Hs00361186_m1 |
| IGFBP5 | Hs00181213_m1 | WNT5A | Hs00998537_m1 |
| KCNK1 | Hs00158428_m1 | AGXT2L1 | Hs01012280_m1 |
| KRT17 | Hs01588578_m1 | HES1 | Hs00172878_m1 |
| KRT19 | Hs01051611_gH | KRT5 | Hs00361185_m1 |
| LAMB1 | Hs01055967_m1 | NPY1R | Hs01001499_g1 |
| LOXL2 | Hs00158757_m1 | PTEN | Hs02621230_s1 |
| MAGEA3 | Hs00366532_m1 |  |  |
| MAPT | Hs00902194_m1 |  |  |
| MLPH | Hs00225445_m1 |  |  |
| PDGFRB | Hs01019589_m1 |  |  |
| PGF | Hs00182176_m1 |  |  |
| PITX2 | Hs04234069_mH |  |  |

**Supplemental Table I.** Gene Symbols and TaqMan Primer-Probes used for qRT-PCR analysis of 46-gene panel.

| **Gene** | **# Positive (%)** | **ER+** | **ER-** | **N+** | **N-** | **M+** | **M-** |
| --- | --- | --- | --- | --- | --- | --- | --- |
| PLAT | 59 (84%) | 25 (89% ) | 34 (80% ) | 23 (76% ) | 33 (91% ) | 19 (90% ) | 40 (81% ) |
| STEAP | 43 (61%) | 17 (60% ) | 26 (61% ) | **15 (50% )** | **26 (72% )** | 11 (52% ) | 32 (65% ) |
| GRB7 | 39 (55%) | **11 (39% )** | **28 (66% )** | 16 (53% ) | 22 (61% ) | 15 (71% ) | 24 (48% ) |
| KCNK1 | 39 (55%) | 16 (57% ) | 23 (54% ) | 17 (56% ) | 20 (55% ) | 11 (52% ) | 28 (57% ) |
| SIP1 | 29 (41%) | 13 (46% ) | 16 (38% ) | 11 (36% ) | 17 (47% ) | 9 (42% ) | 20 (40% ) |
| IGFBP5 | 28 (40%) | 9 (32% ) | 19 (45% ) | 11 (36% ) | 16 (44% ) | 8 (38% ) | 20 (40% ) |
| MLPH | 26 (37%) | 12 (42% ) | 14 (33% ) | 12 (40% ) | 11 (30% ) | **12 (57% )** | **14 (28% )** |
| SCUBE2 | 25 (35%) | **14 (50% )** | **11 (26% )** | 9 (30% ) | 14 (38% ) | 7 (33% ) | 18 (36% ) |
| CDH3 | 22 (31%) | 7 (25% ) | 15 (35% ) | 9 (30% ) | 12 (33% ) | 8 (38% ) | 14 (28% ) |
| HSPB7 | 21 (30%) | 9 (32% ) | 12 (28% ) | 7 (23% ) | 10 (27% ) | 6 (28% ) | 15 (30% ) |
| SNAIL1 | 21 (30%) | 8 (28% ) | 13 (30% ) | 11 (36% ) | 10 (27% ) | 5 (23% ) | 16 (32% ) |
| CAV1 | 20 (28%) | 7 (25% ) | 13 (30% ) | 8 (26% ) | 11 (30% ) | 7 (33% ) | 13 (26% ) |
| LAMB1 | 17 (24%) | **10 (35% )** | **7 (16% )** | 9 (30% ) | 7 (19% ) | 4 (19% ) | 13 (26% ) |
| PGF | 17 (24%) | 7 (25% ) | 10 (23% ) | 9 (30% ) | 7 (19% ) | 4 (19% ) | 13 (26% ) |
| WNT5A | 17 (24%) | **10 (35% )** | **7 (16% )** | 4 (13% ) | 10 (27% ) | 4 (19% ) | 13 (26% ) |
| S100A3 | 15 (21%) | 4 (14% ) | 11 (26% ) | 5 (16% ) | 10 (27% ) | 6 (28% ) | 9 (18% ) |
| SLIT2 | 14 (20%) | 7 (25% ) | 7 (16% ) | 6 (20% ) | 7 (19% ) | 5 (23% ) | 9 (18% ) |
| PDGFRB | 10 (14%) | 5 (17% ) | 5 (11% ) | 4 (13% ) | 5 (13% ) | 3 (14% ) | 7 (14% ) |
| CCND1 | 9 (12%) | 3 (10% ) | 6 (14% ) | 5 (16% ) | 3 (8% ) | 2 (9% ) | 7 (14% ) |
| SMO | 9 (12%) | 2 (7% ) | 7 (16% ) | 2 (6% ) | 7 (19% ) | 4 (19% ) | 5 (10% ) |
| IGFBP4 | 9 (12%) | 3 (10% ) | 6 (14% ) | 3 (10% ) | 6 (16% ) | 4 (19% ) | 5 (10% ) |
| ERBB2 | 8 (11%) | 2 (7% ) | 6 (14% ) | 5 (16% ) | 3 (8% ) | 4 (19% ) | 4 (8% ) |
| FGFR4 | 7 (10%) | 4 (14% ) | 3 (7% ) | 2 (6% ) | 5 (13% ) | 2 (9% ) | 5 (10% ) |
| PTCH1 | 6 (8%) | 3 (10% ) | 3 (7% ) | 2 (6% ) | 4 (11% ) | 1 (4% ) | 5 (10% ) |
| EPCAM | 6 (8%) | **0 (0% )** | **6 (14% )** | 2 (6% ) | 4 (11% ) | 3 (14% ) | 3 (6% ) |
| PITX2 | 5 (7%) | 1 (3% ) | 4 (9% ) | 3 (10% ) | 2 (5% ) | 3 (14% ) | 2 (4% ) |
| PTPRN2 | 5 (7%) | 3 (10% ) | 2 (4% ) | 3 (10% ) | 2 (5% ) | 2 (9% ) | 3 (6% ) |
| GLI3 | 4 (5%) | 2 (7% ) | 2 (4% ) | 1 (3% ) | 3 (8% ) | 1 (4% ) | 3 (6% ) |
| AGXT2L1 | 3 (4%) | 2 (7% ) | 1 (2% ) | 3 (10% ) | 0 (0% ) | **3 (14% )** | **0 (0% )** |
| SRC | 3 (4%) | 1 (3% ) | 2 (4% ) | 0 (0% ) | 3 (8% ) | 0 (0% ) | 3 (6% ) |
| SCGB2A2 | 2 (2%) | 1 (3% ) | 1 (2% ) | 1 (3% ) | 0 (0% ) | 0 (0% ) | 2 (4% ) |
| TWIST1 | 2 (2%) | 1 (3% ) | 1 (2% ) | 2 (6% ) | 0 (0% ) | 2 (9% ) | 0 (0% ) |
| KRT5 | 1 (1%) | 0 (0% ) | 1 (2% ) | 0 (0% ) | 1 (2% ) | 0 (0% ) | 1 (2% ) |
| SNAI2 | 1 (1%) | 1 (3% ) | 0 (0% ) | 0 (0% ) | 1 (2% ) | 0 (0% ) | 1 (2% ) |

**Supplemental Table II.** Frequency of gene transcript positivity in 70 breast cancer patient BM samples, dichotomized by estrogen receptor status of the primary tumor (ER+ vs. ER-), presence of lymph node metastasis (N+ vs. N-), and development of distant metastatic disease (M+ vs. M-). Numbers in bold are significantly different (p < 0.05 Fishers Exact Test) but are not significant when corrected for multiple comparisons (**FDR**).
